# Complement pathway: Risk molecular pattern of cardiovascular diseases in patients with diabetic nephropathy

**DOI:** 10.5937/jomb0-56221

**Published:** 2025-07-04

**Authors:** JunFei Liu, XiaoPing Qian, TaoXia Wang, XiaoLi Liu, WeiGang Liu, LiJie Wang, HuiFang Zhang, Li Han, GuiYing Li, XiaoJuan Yu

**Affiliations:** 1 Affiliated Hospital of Hebei University of Engineering, Department of Nephrology, The Key Laboratory of Basic Research on Blood Purification Application in Hebei Province, Handan City, Hebei Province, China; 2 Affiliated Hospital of Hebei University of Engineering, Department of Nutrition, Handan City, Hebei Province, China

**Keywords:** complement pathway, diabetic nephropathy, cardiovascular events, complications, sistem komplementa, dijabetička nefropatija, kardiovaskularni događaji, komplikacije

## Abstract

**Background:**

Among individuals with diabetes mellitus, diabetic nephropathy (DN) is a common microvascular complication. As renal dysfunction progresses in DN patients, the risk of cardiovascular disease (CVD) significantly increases. The current effective treatment of DN and CVD demands identifying and managing their risk factors.

**Methods:**

Patients diagnosed with DN from October 2022 to October 2023 were selected for cross-sectional study and divided into DN group (n=58) and DN/CVD group (n=40) based on the presence of CVD. Univariate analysis was conducted using clinical data, and statistically significant independent variables were analysed through multivariate Logistic regression to identify independent factors influencing CVD in DN patients. A regression model was developed to examine the non-linear relationship between C1q, UA, CRP, and the risk of CVD. The receiver operating characteristic curve was used to analyse the predictive efficacy of the indicators.

**Results:**

When elevated, UA and C1q were independent factors for CVD. A linear relationship existed between UA and C1q and the risk of CVD in DN patients. C1q showed better predictive performance.

**Conclusions:**

As UA and C1q levels rise, the risk of CVD in DN patients significantly increases. In DN patients, UA and C1q are associated with CVD development and progression, offering some supportive evaluation value for the patient's condition.

## Abstract (sr)

**Uvod:**

Dijabetička nefropatija (DN) predstavlja čestu mikro - vaskularnu komplikaciju kod osoba sa dijabetes melitusom. Kako bubrežna disfunkcija napreduje kod pacijenata sa DN, rizik od kardiovaskularnih bolesti (CVD) se značajno poveća. Efikasno lečenje DN i CVD zahteva identifikaciju i upravljanje faktorima rizika.

**Metode:**

Pacijenti sa dijagnozom DN, u periodu od oktobra 2022. do oktobra 2023, uključeni su u studiju preseka i podeljeni u DN grupu (n=58) i DN/CVD grupu (n=40) na osnovu prisustva CVD. Univarijantna analiza je sprovedena korišćenjem kliničkih podataka, a statistički značajne nezavisne varijable su analizirane putem multivarijantne logističke regresije radi identifikacije nezavisnih faktora koji utiču na razvoj CVD kod DN pacijenata. Razvijen je regresioni model za ispitivanje nelinearne veze između C1q, UA, CRP i rizika od CVD. ROC kriva je korišćena za analizu prediktivne efikasnosti indikatora.

**Rezultati:**

Povišeni nivoi UA i C1q pokazali su se kao nezavisni faktori za razvoj CVD. Ustanovljena je linearna povezanost između UA i C1q i rizika od CVD kod pacijenata sa DN. C1q je pokazao bolju prediktivnu vrednost.

**Zaključak:**

Sa porastom nivoa UA i C1q, rizik od CVD kod pacijenata sa DN značajno se povećava. UA i C1q su povezani sa razvojem i progresijom CVD kod DN pacijenata, pružajući određenu podršku u proceni stanja pacijenata.

## Introduction

Diabetic nephropathy (DN) is a prevalent microvascular complication among individuals with diabetes mellitus (DM). This condition ranks as the most pervasive secondary kidney disorder, affecting 30–40% of diabetic individuals around the world and is the leading cause of end-stage renal disease (ESRD) [1]. Chronic kidney disease (CKD) patients often experience cardiovascular disease (CVD). With the decrease in glomerular filtration rate, the risk of CVD is increasing. In DN patients, CVD occurs at a higher rate than in the general population and is the leading cause of death among ESRD patients undergoing maintenance dialysis [2]. Evaluating the risk factors for CVD in patients with DN is significant for clinical care and minimising mortality.

DN is closely related to metabolic abnormalities, hemodynamics, oxidative stress, inflammatory response, and autophagy [3]. Recent research indicates that inflammation and immune response are central to the pathogenesis of DN [4]. Involving immune and inflammatory responses, the complement system is a protein network essential for the body’s defence against infections, inflammation, and immune response [5]. Complement 1q (C1q) is the first component of the complement system C1 and is the initiating factor for activating the classical complement pathway [6]. Previous investigations have revealed that the relationship between C1q and CVD is controversial. On the one hand, C1q locally recognises pathogens and induces complement activation, removing metabolites without complete complement activation; on the other hand, extensive classical pathway activation can induce pro-inflammatory signals, which may accelerate the progression of CVD [7] [8]. C1q has a dual function in both coronary heart disease (CHD) and heart failure. On the one hand, it activates inflammation through complement reaction and promotes disease progression. On the other hand, it acts as a safeguard in the early stages of plaque development in CHD [9]. In cases with C1q deposition, interstitial fibrosis, tubular atrophy, interstitial inflammation, and vascular lesions are markedly aggravated in DN. C1q damages the renal vascular endothelium and accelerates inflammation progression. Complement deposition in renal histopathology is related to more severe kidney damage in DN patients [10]. According to a retrospective study, patients with glomerular C1q deposition have worse renal survival than those without such deposition [11]. Nonetheless, research on the effect of C1q on CVD risk in DN patients is still lacking.

This study collected and analysed all patients’ general clinical data and complement pathway indicators, explored their relationship with CVD in DN patients, and identified influencing factors of CVD.

## Materials and methods

### Patients

The clinical data of 98 patients with DN admitted to Affiliated Hospital of Hebei University of Engineering from October 2022 to October 2023 were retrospectively analysed. Based on the presence of CVD, patients were divided into the DN/CVD group (40 cases) and the DN group (58 cases). All patients underwent examinations, such as cardiac ultrasound and electrocardiogram, with coronary atherosclerotic heart disease, arrhythmia or heart failure as complications associated with CVD.

### Inclusion criteria

(1) Patients met the diagnostic criteria of the Chinese guidelines for diagnosis and treatment of diabetic kidney disease (2021 edition), which is in line with the definition of DN by the World Health Organization; (2) Patients were 18 years old; (3) Estimated glomerular filtration rate (eGFR) was 20 μg/min.

### Exclusion criteria

(1) patients with type 1 DM, pregnancy, and special types of DM; (2) patients with acute complications of DM, such as diabetic ketoacidosis and hyperosmolar hyperglycemia; (3) patients with primary glomerular and tubular diseases; (4) patients with acute and chronic pancreatitis; (5) patients with severe liver dysfunction; (6) patients with acute infection; (7) patients with familial hypertriglyceridemia.

### Diagnostic criteria

Based on the American Diabetes Association guidelines and earlier research, a diagnosis of DM is given to those who satisfy any of these conditions: (1) previous diagnosis by medical professionals; (2) Fasting blood glucose 7.0 mmol/L; (3) glycosylated haemoglobin greater than or equal to 6.5%; (4) patients undergoing hypoglycemic treatment currently. The eGFR was calculated using the CKD Epi demiology Collaboration. Patients with urinary albumin-to-creatinine ratio 30 μg/mg and/or eGFR<60 mL/(min·1.73 m^2^) were diagnosed as DN.

CVD diagnostic criteria: Typical manifestations such as chest tightness, shortness of breath, cyanosis, or chest pain were present. Based on electrocardiogram and echocardiography findings, patients with coronary atherosclerotic heart disease, arrhythmia, or heart failure were identified as having CVD [12].

### General data collection

The patients’ general data were collected, including gender, age, body mass index (BMI), duration of DM, smoking history, drinking history, and history of hypertension.

### Laboratory indicators

Before blood sampling, the patients were on a light diet and fasted for over 8 hours. The elbow venous blood and 24-hour urine were collected the next morning. Blood urea nitrogen (BUN) was measured using an automatic biochemical analyser (Beckman Coulter, IMMAGE800) with matching reagents. Serum creatinine (SCr), uric acid (UA), aspartate transaminase (AST), alanine aminotransferase (ALT), total cholesterol (TC), triglycerides (TG), high-density lipoprotein (HDL), low-density lipoprotein (LDL), and C-reactive protein (CRP) were measured. Glycosylated haemoglobin A1c (HbA1c) was determined by high-performance liquid chromatography, and fasting blood glucose (FBG) was analysed by hexokinase method using the automatic biochemical analyser (Bio-Rad, AU5800). C1q was determined by immunoturbidimetry.

### Statistical analysis

SPSS 26.0 was used for statistical analysis. The measurement data were tested for normality. Data conforming to the normal distribution were expressed as mean±standard deviation (SD) and compared using the student t-test. Non-normally distributed data were represented by median and interquartile range M (Q25–Q75) and compared by Wilcoxon signed rank sum test. Count data were compared by chi-square test. Multivariate Logistic regression analysis included Independent variables with statistically significant differences in univariate analysis (P<0.05). The odds ratio (OR) was calculated. A sample regression model was constructed to test whether there was a non-linear relationship between C1q, UA, CRP and CVD risk. The area under the curve (AUC), confidence interval, sensitivity, specificity and cut-off value were obtained by receiver operating characteristic (ROC) curve analysis. *P*<0.05 indicated a significant difference.

## Results

### Clinical general data

All patients were divided into the DN group (n=58) and the DN/CVD group (n=40) based on echocardiography and electrocardiogram. The probability of CVD in DM patients was 40.82%. Patients in the DM group were aged 55–62, with an average age of 57.01±5.27 years. In the DN/CVD group, patients were aged 52–63, with an average age of 58.00±5.82 years old. There was no significant difference in age, BMI, past medical history, DM course, and blood routine examination (AST, ALT, FBG, etc.) between the two groups (*P*>0.05). HbA1c, UA, CRP and C1q in the DN/CVD group were significantly higher than those in the DN group (all *P*<0.05) (Table 1).

**Table 1:** Comparison of clinical general data between DN group and DN/CDV group. Data were expressed as mean ± standard deviation or number of cases (%). Continuous variables were tested using the student t-test, and count data was tested using the chi-square test. P<0.05 was considered statistically significant.<br>Abbreviations: DN, diabetic nephropathy; CVD, cardiovascular disease; BMI, body mass index; DM, diabetes mellitus; AST, aspartate transaminase; ALT, alanine aminotransferase; ALB, albumin; FBG, fasting blood glucose; WBC, white blood count; HDL, high-density lipoprotein; LDL, low-density lipoprotein; TC, total cholesterol; TG, terrogram; HbA1c, glycated haemoglobin; BUN, blood urea nitrogen; UA, uric acid; Scr, serum creatinine; CRP, hypersensitive C-reactive protein; C1q, complement 1q.

Characteristics	DN group<br>(n=58)	DN/CVD group<br>(n=40)	*P*-value
Gender (male)	32 (55.17%)	23 (57.50%)	0.41
Age (year)	57.01±5.27	58.00±5.82	0.38
BMI (kg/m^2^)	24.39±0.49	24.55±0.69	0.17
Smoking history	21 (36.21%)	14 (35.00%)	0.28
Drinking history	28 (48.28%)	17 (42.50%)	0.06
Hypertension	12 (20.69%)	8 (20.00%)	0.56
DM course	9.67±5.54	11.78±6.86	0.1
AST (U/L)	20.55±2.52	21.34±4.16	0.25
ALT (U/L)	22.89±3.36	21.51±4.34	0.08
ALB (g/L)	42.51±5.06	40.68±4.36	0.07
FBG (mg/dL)	168.66±32.15	174.74±40.26	0.41
WBC (10^9^/L)	6.83±1.54	7.11±2.07	0.44
HDL (mmol/L)	1.21±0.81	1.25±0.66	0.8
LDL (mmol/L)	2.33±1.50	2.37±1.02	0.88
TC (mmol/L)	4.72±1.2	4.46±1.05	0.27
TG (mmol/L)	1.74±0.91	2.10±1.05	0.07
HbA1c (%)	9.95±1.59	10.97±1.74	0
BUN (mmol/L)	9.50±5.00	12.04±8.05	0.06
UA (μmol/L)	405.70±59.36	439.20±64.28	0.01
Scr (μmol/L)	68.61±19.52	65.52±14.92	0.4
CRP (mg/L)	10.35±1.88	11.38±1.71	0.01
C1q (mg/dL)	10.15±2.15	12.67±2.29	<0.01

### Multivariate Logistic regression analysis of CVD in patients with DN

A multivariate logistic regression model was created using HbA1c, UA, CRP, and C1q as independent variables, as these factors showed significant differences (*P*<0.05) in Table 1. HbA1c (OR=1.307, P=0.136) was not an independent factor for CVD in patients with DN. Elevated levels of UA, CRP and C1q were independent risk factors for CVD. Among them, C1q was the most significant, with an OR value of 1.569 (*P*<0.001), followed by UA (OR=1.010, *P*=0.024) and CRP (OR=1.579, *P*=0.005) (Table 2).

**Table 2:** Multivariate Logistic regression model was used to analyze the risk factors of CVD in DN patients. Abbreviations: DN, diabetic nephropathy; CVD, cardiovascular disease; UA, uric acid; HbA1c, glycated haemoglobin; CRP, hypersensitive C-reactive protein; C1q, complement 1q; OR, odds ratio; CI, confidence interval. P < 0.05.

Indices	OR	95%CI	P value
UA	1.01	1.001–1.018	0.024
HbA1c	1.307	0.920–1.856	0.136
CRP	1.579	1.148–2.172	0.005
C1q	1.569	1.245–1.976	<0.001

### Linear relationship between UA, CRP and C1 q and CVD risk in DN patients

**Figure 1:**
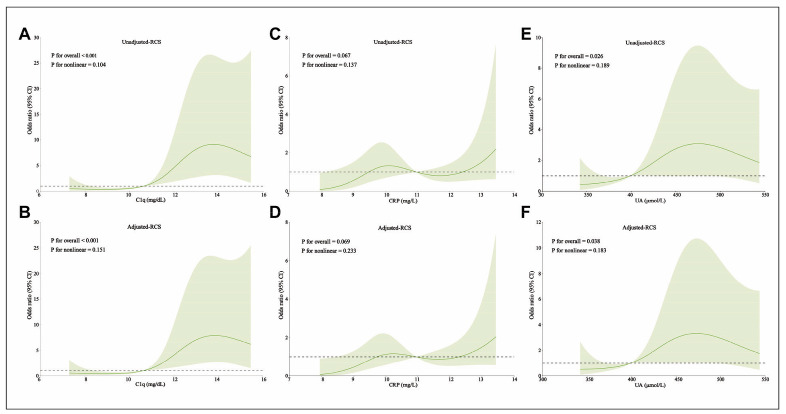
Comparison of important perioperative time indicators.<br>(A: time of recovery of heartbeat; B: postoperative recovery time; C: postoperative extubation time; D: length of ICU stay; E: length of hospital stay; Note: # as against controls, P<0.01)

A restricted cubic spline model was employed to depict the relationship between UA, CRP, C1q, and the risk of CVD in DN patients. After adjusting for the confounding factor HbA1c, UA (P_for nonlinear_=0.183), CRP (P_for nonlinear_=0.233), and C1q (P_for nonlinear_=0.151) showed a linear relationship with the risk of CVD in patients with DN. UA (P_for overall_=0.038) and C1q (P_for overall_<0.001) were associated with CVD in patients with DN, while CRP (P_for overall_=0.069) was not associated with CVD in patients with DN. With the increase of UA and C1q levels, the risk of CVD in the DN group was significantly increased (Figure 1).

### ROC curve results of UA and C1q in predicting CVD in patients with DN

**Figure 2:**
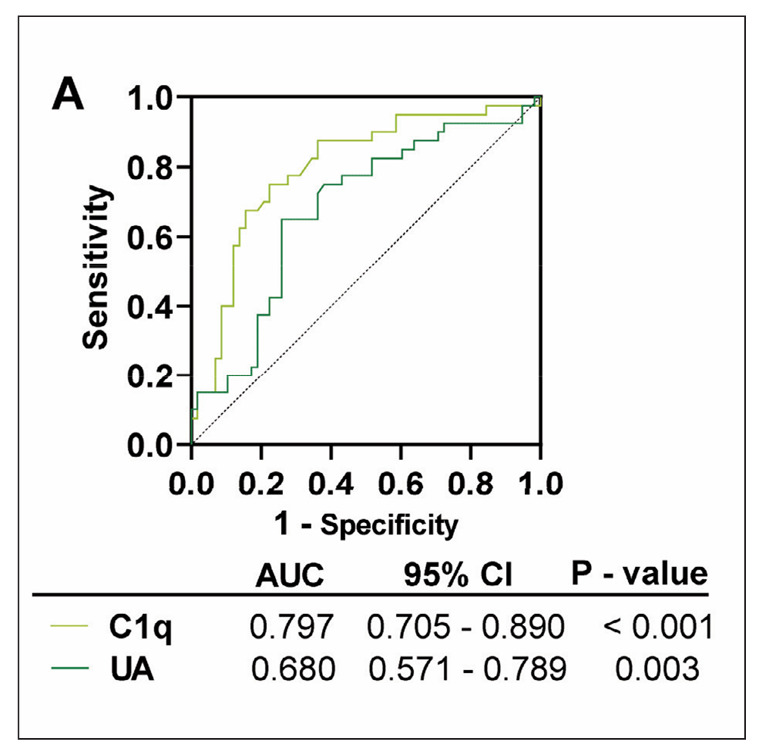
ROC analysis to evaluate the predictive efficacy of UA and C1q on CVD in patients with DN, P<0.05.

The ROC was plotted to further examine the predictive power of UA and C1q on CVD in patients with DN, with the DN/CVD group as the positive sample and the DN group as the negative sample. Both UA and C1q showed good predictive performance. The AUC of C1q was 0.797 (95%CI=0.705-0.890). With a cut-off value of C1q greater than 11.44 mg/dL, the sensitivity and specificity for predicting CVD were 75.00% and 77.59%, respectively. The AUC for UA was calculated to be 0.680, with a 95% confidence interval of 0.571 to 0.789. At a UA level exceeding 412.40 μmol/L, the sensitivity and specificity for predicting CVD were 65.00% and 74.14%, respectively (Table 3, Figure 2).

**Table 3:** ROC curve results of UA and C1q in predicting CVD in DN patients. Abbreviations: DN, diabetic nephropathy; CVD, cardiovascular disease; UA, uric acid; C1q, complement 1q; PPV, positive predictive value; NPV, negative predictive value.

Indices	Cut-off	Youden index	Sensitivity (%)	Specificity (%)	PPV (%)	NPV (%)
UA (mmol/L)	412.4	0.392	65	74.14	62.71	72.92
C1q (mg/dL)	11.44	0.526	75	77.59	69.13	81.31

## Discussion

The study found that elevated UA and C1q levels independently raised the risk of CVD in DN patients, with C1q being the most significant predictor. There was a linear relationship between UA, C1q, and the risk of CVD in DN patients, with a noted correlation. The involvement of complement C1q in CVD in DN patients is evident.

The inflammatory response in atherosclerosis heavily involves the immune system, with C1q being a major connection between innate and specific immune responses. Some scholars have clearly pointed out that serum C1q level in patients with CVD is significantly increased, and low C1q level is a protective factor for CHD [13]. However, it has also been reported that the level of serum C1q in patients with CHD is lower, and a low level of C1q is a risk factor for CHD [14] [15]. These inconsistent conclusions may be related to the different development stages of CVD, the different measurement methods of serum complement C1q, and the different sample sizes. In a seven-year study involving 547 healthy individuals, HERTLE and colleagues discovered that both elevated and reduced C1q levels were linked to a 2 to 2.5 times higher risk of CVD. C1q’s non-linear relationship with long-term cardiovascular pathology reveals its dynamic development with CVD, indicating it cannot predict future cardiovascular risk changes at the current serum level [16]. Goulielmos et al. revealed that complement C1q was associated with an increased risk of susceptibility in patients with type 2 DM [17]. Kaye S et al. [18] found that C1q was upregulated in obese people. Research has largely confirmed a potential association between the complement system and DN, concentrating mainly on the MBL pathway. But, recently, some scholars have focused on the classical pathway and DN. C1q deposition is found in the glomerular capillary wall and renal tubular basement membrane of DN patients [19]. This points to C1q being vital in disease development and kidney injury in those with DN. This study explored CVD risk factors in DN patients and found that serum C1q levels in DN/CVD patients increased significantly. This suggests that C1q, as a key component of the complement system, can be used as an early predictive marker for developing CVD in patients with DN. In clinical practice, regular testing of C1q levels and dynamic assessment of patients’ cardiovascular risk can help create personalised treatment strategies for timely intervention of patients’ CVD risk. In addition, incorporating C1q testing into the routine management of DN patients will effectively improve the comprehensiveness of the actual monitoring of patients.

This study also found that UA had a predictive effect on CVD in DN patients. Serum UA concentration >6.3 mg/dL is an independent risk factor for CKD stage progression [20]. UA is negatively correlated with the function of islet B cells in DM patients [21]. UA is a significant risk factor for cardiovascular and cerebrovascular diseases, as it can engage in oxidation reactions and interact with lipoprotein cholesterol to form thrombi, generate oxygen-free radicals, and trigger atherosclerotic damage [22]. In addition, the abnormal increase of UA level may result in substantial deposition of urate crystals within the body, stimulate leukocyte adhesion to endothelial cells, cause vascular inflammation, and heighten the risk of CVD [23]. The findings showed that elevated UA levels led to a significant increase in CVD risk in DN patients, suggesting UA could predict CKD risk in DN patients.

This study identified complement-related molecular markers, which can help predict the risk of CVD in DN patients earlier and provide a basis for developing new therapies. In addition, we further demonstrated a linear relationship between UA and C1q and the risk of developing CVD in DN patients. This provides a foundation for future exploration of the complement pathway and the mechanism of CVD in DN patients. However, this study is limited by the absence of a healthy control group and the failure to detect serum complement C1q expression in healthy individuals, which makes it impossible to compare these indicators between the healthy control group and the DN group. In addition, the research participants for this study were limited to those from one hospital, with a small sample size and simple grouping. The follow-up is expected to refine the grouping and undertake more comprehensive studies.

## Conclusion

To sum up, serum complement C1q levels are elevated in patients with DN/CVD. C1q serves as an independent risk factor for CVD in DN patients and has strong predictive power. Our future plans include enlarging the sample size by adding healthy controls and conducting subgroup analyses based on disease severity, age, and other factors to ensure sample diversity and generalizability. Additionally, planning longitudinal studies to observe the variations in complement indices in patients and their correlation to CVD events is an important focus for our upcoming thorough research. In this regard, serum complement C1q is anticipated to be a novel marker for predicting CVD in DN patients, showing a strong linear relationship with CVD in these patients, which will aid in evaluating clinical DN patients with complications and tailoring treatment.

## Dodatak

### Acknowledgements

Not applicable.

### Funding

1. Hebei Provincial Medical Science Research Project: The protective effect of Lycium barbarum polysaccharide on renal ischemia-reperfusion injury by inhibiting NLRP3 inflammasome pathway (No. 20250436)

2. Handan Municipal Science and Technology Bureau Directive Project: Clinical study on the effect of multi-frequency bioelectrical resistance method in evaluating dry weight in hemodialysis patients (No. 19422083011-15)

3. Medical Science Research Projects in Hebei Province (No. 20250961)

### Data available

Data is available from the corresponding author on request.

### Consent to participate

Written informed consent was obtained from each subject.

### Consent to publish

Written informed consent for publication was obtained from all participants.

### Ethics statement

All procedures performed in this study involving human participants followed the ethical standards of the institutional and/or national research committee, along with the 1964 Helsinki Declaration and its later amendments or comparable ethical standards. All subjects were approved by the Affiliated Hospital of Hebei University of Engineering (2024–0026).

### Authors’ contribution

Authors JunFei Liu and XiaoPing Qian contributed equally to this work.

Conceptualisation, JunFei Liu and XiaoPing Qian; data curation, TaoXia Wang and XiaoLi Liu; formal analysis, WeiGang Liu and HuiFang Zhang; investigation, LiJie Wang and Li Han; methodology, GuiYing Li and XiaoJuan Yu; resources, JunFei Liu and XiaoPing Qian; supervision, JunFei Liu and XiaoJuan Yu; writing—original draft preparation, JunFei Liu and XiaoPing Qian; writing – review and editing, XiaoPing Qian and XiaoJuan Yu. All authors have read and agreed to the published version of the manuscript.

### Conflict of interest statement

All the authors declare that they have no conflict of interest in this work.
